# Effects of GnRH agonist treatment on steroidogenesis and folliculogenesis in the ovary of cyclic mice

**DOI:** 10.1186/1757-2215-3-26

**Published:** 2010-11-18

**Authors:** Padmasana Singh, Amitabh Krishna

**Affiliations:** 1 Department of Zoology, Banaras Hindu University, Varanasi 221005, India

## Abstract

**Background:**

GnRH analogs (both agonist and antagonist) have been extensively used for clinical applications, following the discovery of its direct effects on ovary. With regard to the direct actions of GnRH agonist on ovary, conflicting data are reported. The mechanism through which GnRH agonist affect gonadal functions is still obscure. The aim of present study was thus to investigate the effects of treatment with different doses of GnRH agonist, *in vivo and in vitro*, on morphological, physiological and functional changes in the ovary of cyclic mice.

**Methods:**

To find out the effect of GnRH agonist on ovarian activity, cyclic mice were treated with different doses for 8 days and its effect on folliculogenesis (morphological changes in follicle, Estrogen receptor, progesterone receptor), steroidogenesis (circulating progesterone level, StAR, LH-receptor, 3β-HSD), luteinization (Morphology of corpus luteum) and apoptosis (caspase-3, PARP) were observed. To find the in vitro effects of GnRH agonist with or without LH on ovary of mice, changes in the expression of LH-receptor, estrogen receptor, progesterone receptor, 3β-HSD in the ovary and progesterone level in the culture media were investigated.

**Results:**

GnRH agonist treatment produced significant changes in ovarian mass, circulating steroids level and ovarian follicular development, steroidogenesis and apoptosis in the mice. GnRH agonist also caused dose dependent histological changes in follicular development and luteinization. The mice treated with different doses of GnRH agonist showed biphasic effects on steroid synthesis due to its effects on ovarian expression of LH-receptor, StAR, and 3β -hydroxysteroid dehydrogenase proteins. The high dose showed stimulatory effect, whereas pharmacological dose showed inhibitory effect on ovarian follicular development and steroidogenesis. The *in vitro *study generally showed inhibitory effects of GnRH agonist on ovarian activities, which may be reversed by the presence of LH.

**Conclusion:**

Both inhibitory and stimulatory effects found in the present study suggest that GnRH agonist is a versatile tool in the therapy of a variety of gynecological and non-gynecological conditions. This study suggests that the outcome of direct effect of GnRH-ag on ovary depends on LH-responsiveness.

## Background

The decapeptide gonadotropin-releasing hormone (GnRH) is a key regulator of sexual maturation and reproductive functions in mammals. It is secreted from the hypothalamus in a pulsatile manner and stimulates the synthesis and release of gonadotropins, follicular stimulating hormone (FSH) and luteinizing hormone (LH), via specific GnRH receptor located on gonadotrope cells. These gonadotropins in turn, regulate various gonadal functions, such as folliculogenesis, steroidogenesis and apoptosis.

Besides pituitary, the GnRH receptor gene is also expressed in extrapituitary sites including ovarian granulosa cells of rat [1] and human [2-4]. Several lines of evidences suggest that ovarian GnRH receptor transcripts are identical to those found in the anterior pituitary gland [1]. A number of biological responses have been observed upon activation of GnRH receptor in the ovary. The actions of GnRH in ovary vary with the developmental stages of the follicles. GnRH exerts a stimulatory action on preovulatory follicles by inducing oocytes maturation [5] and follicle rupture [6]. On smaller follicles, however, the effects of GnRH are inhibitory in nature as GnRH treatment decreases steroidogenesis and gonadotropes receptor concentration [7]. GnRH may also play a role in the induction of follicular atresia in the rat ovary [8]. Thus, the actions of GnRH within the ovary are diverse.

Many derivatives of GnRH, known as GnRH analogs, have been synthesized in an attempt to develop more potent GnRH compounds for therapeutic use. A large number of structural analogs of GnRH have been synthesized. Both GnRH agonist (GnRH-Ag) and GnRH antagonist (GnRH-Anta) with enhanced biological potency have been developed and studied extensively [9]. Clinically some of these synthetic analogs have been used as an effective treatment of hormone dependent reproductive disorders including infertility, endometriosis, polycystic ovary syndrome (PCOS), precocious puberty, and uterine fibroids etc, whereas others have widely adopted in controlled ovarian hyperstimulation regimes for assisted reproductive techniques [10]. In addition to the therapeutic applications, GnRH analogs are predicted to be used as new generation male and female contraceptives in conjunction with steroid hormone replacement [11,12].

The extensive clinical applications of GnRH analogs have attracted the investigations on direct effect of GnRH-Ag on ovarian activity. However, only limited and conflicting information exists about the effect of GnRH analogs on ovarian morphological and functional features [10,13]. In granulosa cells collected from human for *in vitro *fertilization (IVF) program, some authors found an increased ovarian steroidogenesis induced by GnRH-Ag *in vitro*, which could not be confirmed by others. But some investigators have proposed that inhibitory effect is due to the down regulation of gonadal gonadotropin receptor caused by the increase release of LH secretion [14], while others feel that GnRH and its analogs act directly on the gonad to inhibit ovarian functions [15].

GnRH is now regarded as an important paracrine and autocrine factor in the ovary [16]. However, the mechanism through which GnRH analogs affect gonadal functions in intact cyclic animal is still obscure. There is considerable debate about the utility of GnRH agonist in treatment for infertility, cancer and assisted reproduction technique. Therefore, it has been cautioned that direct effects of GnRH analogs should be explored in more detail prior to their large-scale introduction for the various therapeutic uses. To the best of our knowledge, the mechanism by which GnRH-analogs affects various ovarian activities, such as follicular development, luteinization and steroidogenesis, has not so far been investigated in intact cyclic animal. In order to explore this, the morphological and physiological changes, such as changes in steroid receptors, and steroidogenic and apoptotic factors in the ovary was investigated following *in vivo *and *in vitro *administration of GnRH agonist in the intact cyclic mice.

## Methods

### Animals

All the experiments were conducted in accordance with principles and procedures of animal act, 2002 of Government of India, approved by Departmental Research Committee, Banaras Hindu University. Mice (*Mus musculus*) of Parkes Strain were housed under constant condition of temperature and humidity in a photoperiodically controlled room (L:D 12:12) of our animal house and were provided with commercial food (Pashu Aahar Kendra, Varanasi, India) and tap water ad libitum. Adult (10-12 week old) female mice of nearly similar body mass and exhibiting at least two consecutive 4-5 day cycles were used in this experiment. Regularly cycling mice were randomly allocated into seven groups.

### Treatment

Mice were given single intramuscular injection of different doses (1 μg, 5 μg, or 25 μg/day) of GnRH-Ag ([DTrp^6^, Pro^9^-NEt] GnRH) dissolved in normal saline, daily for 8 days (n = 8-10 in each group). The mice in control group (n = 10) received vehicle only. The treatment with GnRH-Ag was started on proestrus morning for each mouse to maintain the uniformity. The animals were sacrificed by decapitation under mild anaesthesia (anaesthetic ether) within 30 min after last injection. Body mass of each mouse was recorded before killing. Ovaries were dissected out, cleaned from any adhered fat tissue and oviduct, and weighed. The ovary of one side from each animal was snap frozen and kept at -40°C until protein extraction for immunoblots and the contralateral ovary was fixed in bouin's fixative at room temperature for histological evaluation of follicular development. Serum was separated from blood and stored at -20°C until assayed for progesterone (P_4_) and estradiol (E_2_).

### Histology and counting of follicles

Bouin's fixed ovaries were dehydrated, embedded in paraffin wax, sectioned serially (5 μm) and stained with hematoxylin and eosin. The stained sections were then observed under a Nikon Eclipse E200 Microscope (Nikon, Tokyo, Japan). The number of different types of follicles (both preantral and antral excluding primordial) and corpus luteum in the ovaries were counted by examining every fifth serial section of each ovary and then counting the follicles whose plane of section passed through the nucleolus of the oocytes. Preantral and antral follicles were classified according to the number of granulosa cell layers and antrum formation [17]. The numbers of atretic follicles were also counted. Pyknotic cell nuclei in the granulosa cells or changes in the oocytes morphology (e.g. deformed shape, vacuolation, loss of nuclear membrane and/or fragmentation) characterized the follicular atresia [18].

#### *In vitro *study

To determine the direct effects of GnRH-Ag, an *in vitro *study was performed in accordance to Singh et al (2010) [19]. Proestrus ovaries were culture as it contains many maturing follicles as well as to maintain uniformity in the stage of all the ovaries used in every group. Culture medium was a mixture of Dulbecco Modified Eagle's Medium and Ham's F-12 (1:1; v:v) Himedia, Mumbai, India) containing 100 U/ml penicillin, 100 μg/ml streptomycin and 0.1% BSA (Sigma Chemicals Co., St Louis, USA). After initial incubation for 2 h at 37°C culture medium was discarded, and ovaries (one per tube) were finally cultured in 1 ml of medium in a humidified atmosphere with 95% air and 5% CO_2 _for 24 h at 37°C. Ovaries cultured under these conditions appeared healthy and did not show any sign of necrosis after 24 h culture. The treatment of GnRH-Ag (10 ng and 100 ng) was given either alone or together with LH (100 ng/ml). Control groups received 10 μl of phosphate buffered saline/ml of medium/tube. Each treatment and control groups were run in triplicate and the experiment was repeated two times. Ovaries were collected at the end of culture, washed several times with PBS and kept frozen at -40°C for immunoblot study. Media was saved at -20°C until assayed for P_4 _and E_2_.

### Radioimmunoassays for Progesterone (P4) and Estradiol (E2)

Steroids in the serum and culture medium were measured directly by radioimmunoassay using commercial kits (Immunotech, Marseille, France). Assays for P4 and E2 in the serum/culture medium were performed with 50 μl and 100 μl respectively, as per manufacturer's instructions. Bound radioactivity was measured for one minute in Gamma Counter (Beckman, Geneva, Switzerland). Standard, zero tubes and blank tubes were run in parallel with the samples. Intra assay coefficient of variation for all the assays were less than 12%.

### Immunoblotting

Western blotting was performed in accordance with Singh et al (2010) [19]. 10% homogenate of ovaries was prepared. Equal amount of protein (15 to 50 μg) as determined by Lowry's method [20] was loaded on SDS-PAGE (8-12%) for electrophoresis and transferred electrophoretically to polyvinylidene difluoride membrane (PVDF, Immobilon-P; Millipore, Bedford, MA, USA). Blotted membranes were blocked and incubated with appropriate dilution of primary antibodies (LH-receptor and progesterone receptor at a dilution of 1:1500; StAR at a dilution of 1:2000; 3β-HSD and estrogen-receptor-α at a dilution of 1:500; Caspase-3 and PARP at a dilution of 1:1000. Membranes were washed with PBS-Tween 20 buffer and then incubated for 30 min either with anti-rabbit or anti-mouse IgG-horse radish peroxidase antibody. Immunodetection was performed with enhanced chemiluminescence detection system (Bio Rad, Hercules, USA). Experiments were repeated three times with the same result. X-ray films were later scanned and then quantified by densitometry (Image J vs 1.36, NIH, USA). Quality of loading and transfer was assessed with Ponceau S staining and/or β-actin. All immunoblots were normalized to β-actin.

### Statistical analysis

The data were analyzed by one-way ANOVA followed by Duncan's multiple range post hoc test. The difference were considered significant if P < 0.05 or 0.01.

## Results

### Effect of GnRH-Ag treatment on body and ovarian mass (Table 1)

**Table 1:** Effect of GnRH agonist on body mass, ovarian mass and ovarian follicles/CL of mice

Treatment	Body mass (g)	Ovarian mass (mg)	Type of follicles					
			
			Early preantral	Late preantral	Early antral	Late antral	Atretic	Corpus luteum
Control	28.20 ± 0.70	6.64 ± 0.29	70.00 ± 9.61	4.50 ± 2.17	20.75 ± 2.43	10.00 ± 0.00	13.00 ± 1.96	4.00 ± 0.58
GnRH-Ag (1 μg/day)	28.12 ± 0.47	5.09 ± 0.52*	63.00 ± 3.60	4.34 ± 2.34	27.34 ± 8.37	7.33 ± 3.18	10.67 ± 2.96	3.50 ± 0.28
GnRH-Ag (5 μg/day)	26.00 ± 0.71	4.55 ± 0.39*	45.00 ± 9.29	4.34 ± 1.34	12.34 ± 3.84	1.00 ± 0.58*	17.00 ± 5.19*	7.34 ± 0.49*
GnRH-Ag (25 μg/day)	26.50 ± 0.50	3.87 ± 0.26*	51.00 ± 13.45	8.34 ± 1.45*	9.34 ± 5.84	2.00 ± 0.58*	25.00 ± 1.00*	3.34 ± 1.45

Values are expressed as Mean ± S. E. M.

*Values are significantly different (p < 0.05) by Duncan's multiple range test when compared with the control.

No significant change in the body mass was observed while ovarian mass were reduced significantly (P < 0.05) with all the doses of GnRH-Ag after 8 days of treatment as compared with controls.

### Effect of GnRH-Ag treatment on Reproductive cyclicity

Vaginal cytology in control mice showed a regular 4-5 day estrous cycle during the period of the experiment. GnRH-Ag treated mice showed no marked changes in vaginal cytology after 8 days of treatment.

### Effect of GnRH-Ag treatment on ovarian histology and follicular development (Table 1; Figure 1)

**Figure 1:**
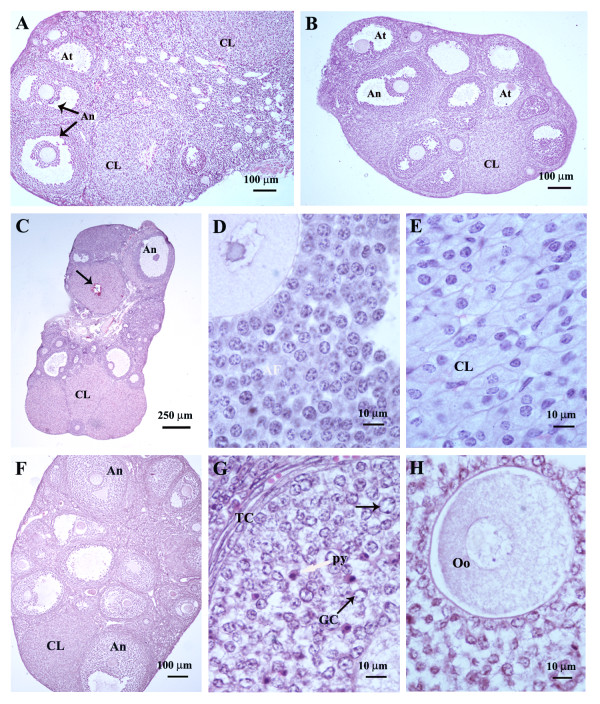
**Transverse sections of the ovaries (stained with haematoxylin-eosin) of cyclic mice showing histological changes following administration of different doses of GnRH-Ag**: (A) The ovary of control mice showing normal corpus luteum (CL), healthy antral follicles (An) and a few atretic follicle (At); (B) ovary of the mice treated with 1 μg/day GnRH-Ag showing many healthy antral follicles (An), a few atretic follicles (At) and a few corpus luteum (CL); (C), (D) & (E) ovary of mice treated with 5 μg/day GnRH-Ag; (C) note the presence of many normal corpus luteum (CL), a few haemorrhagic corpus luteum and antral follicle (An); (D) normal antral follicle (An); (E) normal corpus luteum (CL); (F), (G) & (H) ovary of the mice treated with 25 μg/day of GnRH-Ag; (F) ovary showing many antral follicles (An) but a few corpus luteum (CL); (G) & (H) section of the ovary showing abnormal antral follicle. Granulosa cells (GCs) showing enlarged and vacuolated nuclei (black arrow), a few pyknotic nuclei (py) and thin and regressed theca cells (TC) layers. The oocytes (Oo) appeared highly vacuolated.

The mice treated with GnRH-Ag showed smaller ovaries, as was also apparent by their mass (Table 1). Total numbers of healthy and atretic follicles in the GnRH-Ag treated ovaries varies significantly and they revealed a marked variation in the pattern of follicular development, ovulation and luteinization. The ovaries of control mice showed healthy and large corpus luteum together with numerous small and medium sized antral follicles (Figure 1A). The mice treated with the low dose (1 μg) of GnRH-Ag showed many healthy and atretic antral follicles and corpus luteum in the ovary (Figure 1B). The high dose (5 μg) of GnRH-Ag showed stimulatory effects on the ovary. The ovaries showed a significant increase in the number and size of corpora lutea as compared with the control (Figure 1C). These newly formed corpora lutea contained morphologically healthy luteal cells (Figure 1E). A number of healthy antral follicles (Figure 1D) were also seen though their number declined significantly as compared with the control. The number of atretic follicles was also significantly higher in mice treated with high dose of GnRH-Ag as compared with the control. However, the treatment with pharmacological dose of GnRH-Ag (25 μg/day) showed extensive degenerative changes in the ovary in granulosa cells, theca cells and oocytes. The majority of the granulosa cells showed enlarged and vacuolated nuclei but the nuclei of some granulosa cells were condensed like pyknotic cells. The theca cells appeared thin and fibrous (Figure 1G). The oocytes of many preantral and antral follicles showed degenerative changes. The oocytes of these abnormal follicles were extensively vacuolated (Figure 1H). The degenerative effect is less marked in luteal cells as compared with the granulosa cells.

### Effect of *in vivo *treatment of GnRH-Ag on serum steroid concentration and expression of steroid receptors in the ovary (Figure 2)

**Figure 2:**
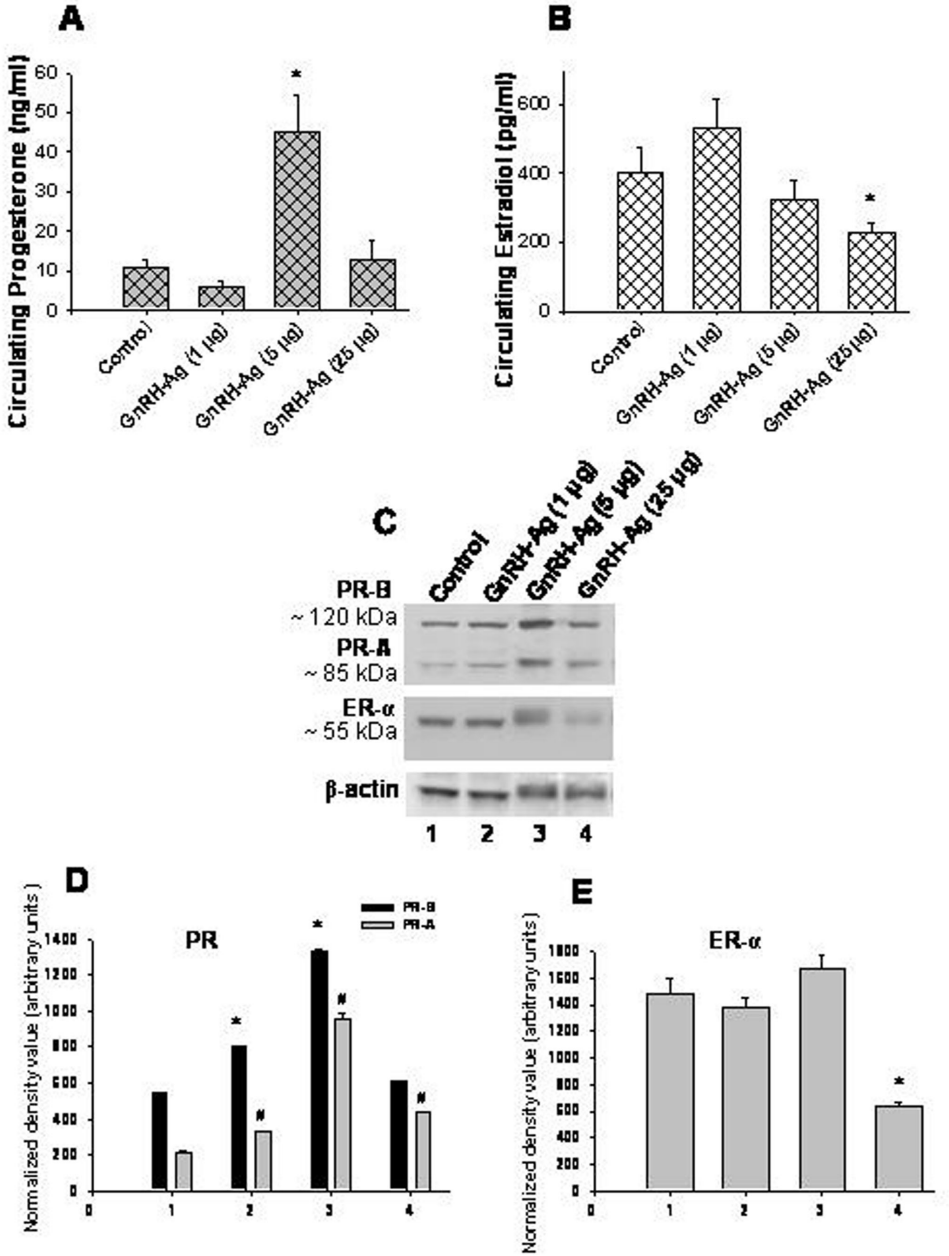
**Effect of in vivo treatment of different doses of GnRH-Ag on circulating steroids and expression of steroid receptors in the ovaries of mice.** A) Circulating progesterone, B) circulating estradiol concentration; C) western blot analyses of PR and ER-α protein. Bar showing densitometric analyses of D) PR and E) ER-α blots (n = 3). Mice treated with normal saline served as control. Values are ± S.E.M. * or # Values are significantly (P < 0.01) different versus control.

Serum P_4 _and E_2 _concentrations of the control and GnRH-Ag treated mice are shown in Figure 2. Particularly, the mice treated with high dose of GnRH-Ag showed significant (P < 0.01) increase in the circulating progesterone level while mice treated with pharmacological dose of GnRH-Ag showed a significant (P < 0.01) decrease in the circulating estradiol concentration as compared with the control mice.

Western blot analysis of progesterone receptor (PR) in the ovary gave two immunoreactive bands at ~85 kDa (PR-A) and ~120 kDa (PR-B) and estrogen receptor-α (ERα) gave single immunoreactive band at ~55 kDa (Figure 2C).

Treatment with low and high doses of GnRH-Ag showed a significant (P < 0.01) increased ovarian expression of both the isoforms of PR while the treatment with pharmacological dose of GnRH-Ag showed significant (P < 0.01) increased expression of only PR-A, but not of PR-B in the ovary (Figure 2D). Treatment with only pharmacological dose of GnRH-Ag showed significant (P < 0.01) decreased expression of ER-α in the ovaries as compared with the control mice (Figure 2E).

### Effect of *in vivo *treatment of GnRH-Ag on expression of LH receptor, steroidogenic acute regulatory protein (StAR) and 3beta-hydroxysteroid dehydrogenase (3β -HSD) proteins (Figure 3)

**Figure 3:**
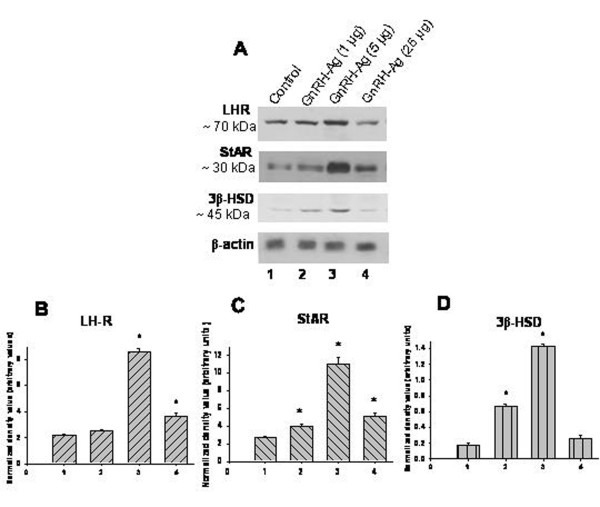
**Effect of in vivo treatment of different doses of GnRH-Ag on LH receptor and steroidogenic markers in the ovaries of mice.** A) Western blot analyses of LH receptor (LH-R), StAR and 3β-HSD proteins. Bar showing densitometric analyses of C) LH-R, D) StAR and E) 3β-HSD blots (n = 3). Mice treated with normal saline served as control. Values are ± S.E.M. * Values are significantly different (P < 0.01) versus control.

Changes in ovarian expression of luteinizing hormone-receptor (LH-R), steroidogenic acute regulatory protein (StAR) and 3β-hydroxysteroid dehydrogenase (3β -HSD) proteins are used as marker of steroidogenic activity in the ovary. Western blot analysis of ovarian LH receptor, StAR, and 3β -HSD proteins in the mice treated with GnRH-Ag showed immunoreactive band at ~70 kDa, ~30 kDa and ~45 kDa respectively (Figure 3A).

The mice treated with the low dose of GnRH-Ag showed no significant variation while high dose treatment showed significantly (P < 0.01) increased expression of LH-R protein in the ovaries as compared with the control. The pharmacological dose treatment of GnRH-Ag showed decline in immunoreactivity for LH-R as compared with the high dose (Figure 3B).

The mice treated with all the three doses of GnRH-Ag showed significantly (P < 0.01) increased expression of StAR protein in the ovary as compared with the control, but the increase is more pronounced in high dose (Figure 3C).

3β-HSD protein increases (P < 0.01) significantly in the ovary of mice treated with low and high doses of GnRH-Ag while pharmacological dose showed no significant variation as compared with the control (Figure 3D).

### Effect of *in vivo *treatment of GnRH-Ag on expression of caspase-3 and poly ADP-ribose polymerase (PARP) proteins (Figure 4)

**Figure 4:**
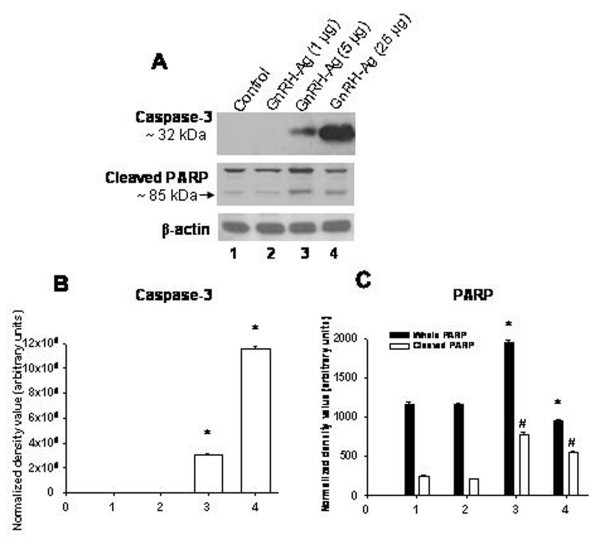
**Effect of in vivo treatment of different doses of GnRH-Ag on apoptosis.** A) Western blot analyses caspase-3 and PARP proteins. Bar showing densitometric analyses of B) caspase-3 and C) PARP blots (n = 3). Mice treated with normal saline served as control. Values are ± S.E.M. * and # Values are significantly different (P < 0.01) versus control.

Changes in ovarian expression of caspase-3 and poly ADP-ribose polymerase (PARP) proteins are used as marker of apoptosis in the ovary. Western blot analysis of caspase-3 gave single immunoreactive band at ~32 kDa while PARP gave two immunoreactive bands at ~116 and ~85 kDa. Immunoreactive band at ~85 kDa corresponds with the cleaved form of PARP (Figure 4A).

The ovaries of control and low dose of GnRH-Ag treated mice showed no marked difference in the expression of caspase-3 proteins. High and pharmacological dose treatment of GnRH-Ag, in vivo, showed a dose dependent increase (P < 0.01) in the expression of caspase-3 in the ovary (Figure 4B).

The control and low dose of GnRH-Ag treated mice showed no significant difference in the immunoreactivity for cleaved form of PARP. But treatment with high and pharmacological doses of GnRH-Ag increases (P < 0.01) cleaved PARP expression in the ovary (Figure 4C).

### In vitro effects of GnRH-Ag on ovarian P_4 _synthesis and LH receptor and 3β HSD proteins expression (Figure 5)

**Figure 5:**
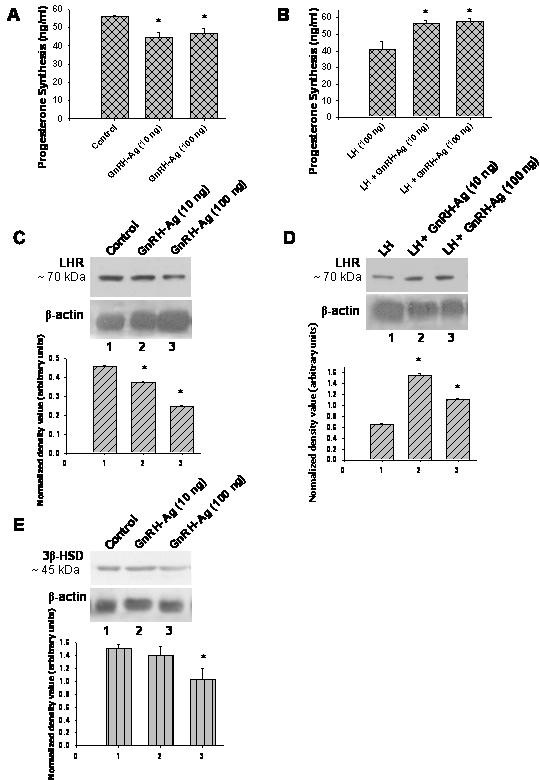
**Effect of in vitro treatment of different doses of GnRH-Ag on progesterone synthesis and western blot analyses of LH-R and 3β-HSD proteins in the ovaries of mice.** Progesterone synthesis by the ovary A) without LH and B) with LH. Western blot analysis of LH-R C) without LH and D) with LH. E) Western blot analysis of 3β-HSD without LH.  Densitometric analyses of the blots are shown in bar graph (n = 3). Values are mean ± S.E.M. * Values are significantly different (P < 0.01) versus control.

The effects of GnRH-Ag with or without LH on steroidogenesis in vitro by the ovaries of mice are shown in Figure 5. Estrogen synthesized, in vitro, by the ovaries of mice was found below the detectable level in both the control and treated groups. The two doses of GnRH-Ag without LH significantly (P < 0.01) suppressed the ovarian progesterone synthesis and LH receptor protein in vitro. Only 100 ng dose of GnRH-Ag significantly (P < 0.01) suppress ovarian 3β-HSD protein expressions.

GnRH-Ag at both the doses significantly (P < 0.01) enhanced LH-induced ovarian progesterone synthesis, in vitro. Both the doses of GnRH-Ag along with LH significantly (P < 0.01) increase LH receptor protein in the ovary as compared to control.

### In vitro effects of GnRH-Ag treatment on ovarian expression of ER-α and PR proteins (Figure 6)

**Figure 6:**
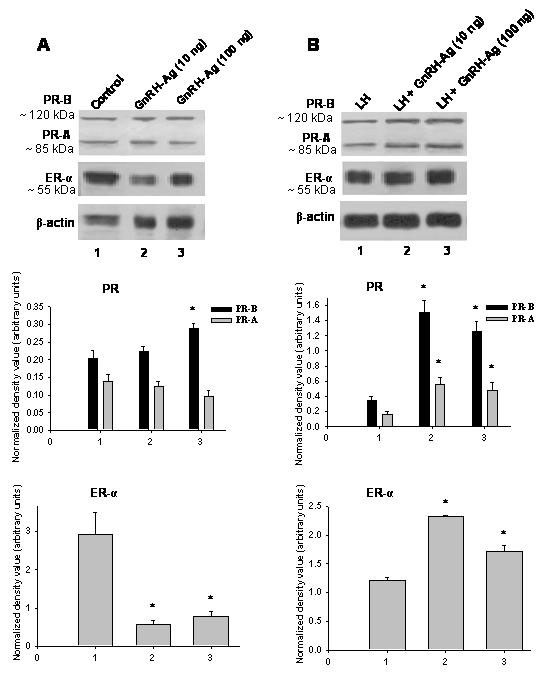
**Effect of in vitro treatment of different doses of GnRH-Ag on steroid receptor expression in the ovaries of mice.** Western blot analyses of PR and ER-α proteins A) without LH and B) with LH. Mice treated with normal saline served as control. Densitometric analyses of the blots are shown in bar graphs (n = 3). Values are ± S.E.M. * and # Values are significantly different (P < 0.01) versus control.

The effect of GnRH-Ag with and without LH on ovarian expression of ER-α and PR proteins in vitro are shown in Figure 6. Both the doses of GnRH-Ag without LH significantly (P < 0.01) suppressed the expression of ER-α but only higher dose increase PR-B protein in the ovaries as compared with the control group. Both the doses of GnRH-Ag together with LH significantly (P < 0.01) enhanced the expression of both ER-α and PR proteins in the ovaries.

## Discussion

The purpose of present study was to evaluate the effects of in vivo and in vitro treatment of GnRH agonist on morphological and physiological changes in the ovaries of intact cyclic mice. Several in vivo and in vitro studies performed in rats have described mostly the antigonadal effect of GnRH analogs [21]. The majority of in vivo study was performed on hypophysectomized rat. Major findings of this study are that in vivo treatment of GnRH-Ag caused both stimulatory and inhibitory effects on ovarian follicular development, ovulation and luteinization in intact cyclic mice. The short term (8 days) treatment with 5 μg per day dose of GnRH-Ag caused stimulatory effects on ovarian steroidogenesis and follicular development. On the other hand, 25 μg per day dose of GnRH-Ag treatment caused inhibitory effects on follicular development and ovulation. The ovaries treated with GnRH-Ag alone in vitro, showed significant decline in progesterone secretion and steroidogenic markers. But when ovaries were treated with GnRH-Ag along with LH, there is increase in progesterone synthesis. This increase in progesterone synthesis is due to increase responsiveness of LH in the presence of GnRH-Ag.

The mice treated with the different doses of GnRH-Ag showed a marked variation in the circulating steroids concentration, luteal morphology and ovarian expression of LH receptor, StAR and 3β-HSD proteins. Treatment with high (5 μg/day) dose of GnRH-Ag showed only a few large antral follicles but showed many newly formed functional corpus luteum in the ovary. These mice also showed significantly high circulating progesterone level, increase expression of LH receptor, StAR and 3β-HSD proteins in the ovary. This can be correlated with healthy luteal morphology suggesting recent ovulation. These observations suggest that treatment with high dose of GnRH-Ag caused stimulatory effects on the ovary, perhaps due to increased gonadotropin release.

The treatment with pharmacological dose of GnRH-Ag showed subnormal luteal morphology and only a marginal increase in the ovarian expression of LH receptor and StAR proteins while no change in 3β-HSD protein expression and circulating progesterone level compared with the control. These observations suggest that the mice treated with pharmacological doses of GnRH-Ag lack functional corpus luteum in the ovary, perhaps due to decreased gonadotropin release. However, the inhibitory action of GnRH analogs on progesterone synthesis has earlier been demonstrated in the ovaries of rat and human [22-24]. It has been reported that short-term GnRH-Ag treatment impairs the progesterone synthesis by directly acting on ovary [25]. Our study showed that the treatment with GnRH-Ag showed both stimulatory and inhibitory effects on steroidogenesis in the ovary. The changes in ovarian steroidogenesis by treatment with GnRH-Ag may be due to their indirect effect on gonadotropin release or direct effects on steroidogenic factors involved in cholesterol transport and/or on the enzyme involved in the steroidogenic pathway.

The mice treated with pharmacological (25 μg/day) dose of GnRH-Ag also showed inhibitory effects on follicular development and ovulation. This was assessed by decreased number of follicles at different stages of development and confirmed by the sharp decline in the circulating E_2 _level and ERα expression in the ovary. It is well known that estrogens are essential for normal follicular growth and are crucial for the survival of ovarian follicles [26,27]. These mice showed morphological sign of regression particularly in the granulosa cells of antral follicles, since GnRH receptor is mainly localized on these cells [3,4]. GnRH-Ag treatment also caused reduction in thecal layer of the antral follicles. This may be probably due to pharmacological dose of GnRH-Ag induced decrease in gonadotropin support to the theca cells and/or indirect suppression of paracrine factors produced by the granulosa cells, which affects thecal development [28]. It is likely that pharmacological dose of GnRH-Ag desensitize pituitary gonadotropes resulting in decreased release of LH and FSH and a decline in ovarian follicular development and steroidogensis [17]. This desensitization phenomenon is extensively applied in clinical medicine for the treatment of wide range of diseases [29].

Although serum gonadotropin levels could not be measured in this study, it is known that moderate dose of GnRH agonist treatment increases LH secretion but FSH remains unaffected [30]. In that case ovulation may be normal but folliculogenesis and selection of dominant follicle is hampered. In our experiment we have found both stimulatory and inhibitory effects of GnRH agonist which dependents on dose of treatment. Treatment with 5 μg dose of GnRH-Ag caused stimulatory effect with stimulatory role in ovulation, while 25 μg dose of treatment failed to induce ovulation or formation of functional CL in the ovary. Both the doses decreased folliculogenesis with decreased number of late antral follicles suggesting low FSH.

Treatment with pharmacological dose of GnRH-Ag significantly suppressed the circulating estradiol concentration when compared with the control. The inhibition of folliculogenesis and the increase in ovarian follicular apoptosis observed in the mice treated in vivo with GnRH-Ag may suggest suppression of gonadotropin secretion resulting in decline in ovarian expression of estrogen receptor and serum estradiol production [28,31,32]. These mice showed presence of several antral follicles, instead of being selected these follicles undergo atresia and produce reduced level of estradiol suggesting decreased gonadotropin secretion [30-32]. It is well known that increase level of gonadotropin stimulation is required for proliferation (growth) and cellular differentiation (steroid synthesis) of the late antral follicles.

The treatment with increasing dose of GnRH-Ag *in vivo *also caused a gradual increase in the number of atretic follicles in the ovaries of cyclic mice. Since follicular atresia is mediated by apoptosis, the effect of GnRH-Ag on apoptotic factors was analyzed in the mice ovary. The results of this study demonstrated that treatment with GnRH-Ag (high and pharmacological dose) *in vivo *showed a significant increase in the ovarian expression of caspase-3 and cleaved PARP proteins in mice. Increase in the expression of caspase-3 and cleaved PARP proteins noticed in the mice treated *in vivo *with GnRH-Ag also showed increase in the number of atretic follicles in the ovary. In an earlier study in rat, GnRH treatment, in vivo, has shown to significantly reduce the mitotic activity of granulosa cells and increase the number of pyknotic cells in the ovarian follicles of hypophysectomized female rats, thus providing evidence for the direct effects of GnRH in follicular atresia [33]. This finding thus support earlier studies that suggest GnRH analogs may enhance follicular atresia and it is by directly stimulating apoptotic factors in the ovary.

The present in vitro study showed suppressive effect of GnRH-Ag alone on ovarian progesterone synthesis. This decrease in progesterone synthesis is associated with decreased LH receptor and 3β-HSD proteins, thus showed inhibitory action of GnRH-Ag on the ovary. However, both the doses of GnRH-Ag together with LH showed increase progesterone synthesis and LH receptor in the ovary. It appears that ovarian GnRH plays a physiological role via local GnRH-gonadotropin axis. GnRH and its receptors have been well demonstrated in the ovary and previous findings showed the physiological role of ovarian GnRH during reproductive cycle of the animal [34,35]. Recently, local production of gonadotropin hormones has also been demonstrated in the ovary of rat [36]. Therefore, the present in vitro study further showed that the ovarian response to GnRH-Ag depends upon the dose of treatment and presence or absence of LH. This finding supports the in vivo study showing high dose of GnRH-Ag caused stimulatory effect on the ovary indirectly by increasing gonadotropin release.

This study further showed that the treatment with GnRH-Ag alone decreases ovarian expression of estrogen receptor protein, but together with LH significantly increases the expression of estrogen and progesterone receptor proteins in the ovary. Since estrogen receptors are required for differentiation of granulosa cells and maturation of dominant follicle and progesterone receptor is required for ovulation [37-40], it may be suggested that GnRH-Ag alone may be responsible for inhibition of follicular development, but in presence of LH, GnRH agonist may be stimulating follicular development and maturation. These effects of GnRH analogs may be modulated by the presence of gonadotropins.

## Conclusions

Treatment of GnRH-Ag in vivo affects follicular development and steroidogenesis in the ovary of cycling mice by acting on ovarian estrogen receptor, progesterone receptor, luteinizing hormone receptor, StAR and 3β-hydroxysteroid dehydrogenase and apoptotic factors. The GnRH-Ag treatment in vitro cause either stimulatory or inhibitory effects on ovary depending upon presence or absence of LH. Thus, this study suggests that the outcome of direct effect of GnRH-Ag on ovary depends on LH-responsiveness.

## Competing interests

The authors declare that they have no competing interests.

## Authors' contributions

PS planned and executed the experiments, responsible for data interpretation and writing of the manuscript, AK helped in planning and supervised the work, participated in data analysis and interpretation. Both the authors have read and approved the final manuscript.
